# Supplementary material for: Are carbon emissions trading and green financial instruments synergistic? -Comprehensive quantitative research based on content analysis
Source: PLoS One. 2024 Mar 7;19(3):e0298601. doi: 10.1371/journal.pone.0298601 (PMC10919601; doi:10.1371/journal.pone.0298601)
Supplement: S1 Data — (ZIP) [file pone.0298601.s001.zip › Data/地级市Z_F工作报告.zip!/║■─╧/│ñ╔│╩╨/└┤╫╘╣1⁄2╓┌║┼í╛┬φ┐╦╩2╛▌═°í┐_2011.docx]

各位代表：

　　现在，我代表市人民政府向大会报告工作，请予审议，并请各位政协委员和其他列席人员提出意见。

　　一、2010年工作及“十一五”简要回顾

　　2010年是形势极为复杂的一年。面对后金融危机的复杂局面和特大洪灾的严峻考验，在市委的坚强领导下，我们坚持又好又快、率先发展，着力建设具有国际视野、人民引以自豪的创业之都、宜居城市、幸福家园，抢抓机遇，科学应对，团结实干，发展好于预期，全面完成了市第十三届人民代表大会第三次会议确定的各项目标任务。

　　----经济实现又好又快发展。实现地区生产总值4500亿元（初步预计数，下同），全社会固定资产投资突破3200亿元，地方财政收入达506.3亿元，分别增长15.5%、32%、27.2%。城镇居民人均可支配收入和农民人均纯收入分别达到21900元和11367元，增长11.5%和20.5%。

　　----抗击特大洪灾取得全面胜利。面对新中国成立以来湘江流域第三大洪水的袭击，全市紧急动员，广大干部群众巡堤、值守、抢险，昼夜奋战，众志成城，严防死守，成功抗击特大洪峰，未垮一库一坝、未溃一堤一垸、未死一人。

　　----交通基础设施建设掀起高潮。续建和新建一批支撑长远发展的重大交通基础设施项目。地铁2号线、营盘路隧道、黄花机场改扩建、京珠复线、长浏高速等重大项目全面建设，湘府路大桥、福元路大桥、南湖路隧道、沪昆高铁、长株潭城际铁路加快推进，地铁1号线、大河西交通枢纽工程开工建设，先导区“六纵八横”路网基本形成。

　　----生态环境建设效果凸显。城区54个排污口实现截污，城市污水处理率提高到90%，曾经污染严重的圭塘河流域水质明显改善；完成天马山、凤凰山和桃子湖“两山一湖”一期整治工程；扬尘污染得到有效治理，城市空气质量优良率92.6%，成功创建全国绿化模范城市。新建乡镇污水处理厂12个，1000个村建立农村垃圾收集处理系统，农村环境卫生明显改善，成为全国农村生态环境保护典型示范城市。

　　----工业发展再上新台阶。全部工业总产值突破5000亿元、增加值突破2000亿元。工程机械产业完成产值1200亿元，成为全市第一个千亿产业集群。新增住友轮胎、美国空气化工等世界500强企业5家，宁乡经开区晋升为国家级开发区。

　　----民生保障持续改善。株树桥引水工程建成通水。生态动物园建成开园。城乡居民最低生活保障标准月均再提高50元和30元。实现城乡居民基本养老保险全覆盖，全市60周岁以上城市无业居民和农民每月享受60元基本养老金。积极争取上级支持，撤销浏永、浏大、宁横、长湘4条二级公路7个收费站点，全面结束长沙普通公路收费通行的历史。群众的幸福感不断增强，连续3年被评为中国十大最具幸福感城市。

　　----城市的知名度和影响力进一步提升。国内外客商纷至沓来，黄花机场国际航线增加到18条，进出港总人数突破1400万人次。与加拿大维多利亚、菲律宾维甘等城市建立友好联系。新增多家银行机构落户长沙。被评为中国（大陆）国际形象最佳城市和2010联合国旅游业发展示范城市。在全国公共文明指数测评中居省会和副省级城市第5位。

　　----政府精细化管理深入推进。加强民主决策和科学决策，市政府常务会议和办公会议研究决定重大事项300多个。优化政府投资项目方案设计，加强可研评审和招投标管理，严格控制超预算投资，核减投资规模23.8亿元；审计政府投资项目978个，审减资金22.6亿元；严格部门预算管理，市本级机关公用经费零增长。用有限的财力支撑了大规模市政基础设施建设和民生保障。

　　一年来，我们着重加大了以下八个方面的工作力度：

　　一是着力推进重大项目建设。紧紧围绕最紧迫、最急需解决的问题，实施重点项目485个，加快基础设施、民生工程和产业建设“三十”重大工程项目建设。“两馆一厅”项目主体工程完工，湘江大道南延线、潇湘大道北段建成通车，湘江综合枢纽工程加快推进，比亚迪、广汽菲亚特、红太阳光伏、蓝思科技等重点产业项目来势喜人。加大项目融资力度，新增上市公司7家，市场直接融资323亿元；直接利用外资22.4亿美元，引进市外境内资金668亿元；新增银行贷款1205亿元。多元化融资为项目建设提供了资金保障。

　　二是着力转变经济发展方式。加快产业结构调整，推进技术改造和自主创新，培育战略性新兴产业，实现高新技术产业总产值2000亿元，增长30%；非公有制经济占地区生产总值的60%；园区规模工业增加值占全市规模工业的53.6%。实施科技重大专项，引进先进适用技术，第四届“科交会”签约251.9亿元，成为国家创新型试点城市。专利授权量突破6000件，获批全国知识产权投融资服务试点城市。推进芙蓉中央商务区建设，成功举办和承办“2010中国?长沙环湘江自行车邀请赛”等重大赛事和节会，努力扩大消费需求，实现社会消费品零售总额1812.6亿元，增长20%。培育发展物流产业，金霞经济开发区被评为中国物流示范基地。实现进出口总额61亿美元、服务外包业务总量245亿元、旅游总收入458亿元，分别增长48%、39.2%和28.7%。落实房地产宏观调控政策，商品房销售面积2000多万平方米，同比增长22.7%。

　　三是着力推进宜居城市建设。坚持以规划引导城市发展，启动城市总体规划修改工作；严格规划管理，坚决查处严重违反规划的行为。加快中心城区提质改造，完成棚户区和工矿棚户区改造115万平方米，东牌楼、大古道巷、氵荣湾镇、丝茅冲、5712厂等棚户区改造项目顺利推进，万达广场、保利南湖广场等大型城市综合体相继建设。启动“城中村”改造试点。深入创建全国文明城市和人民满意城市，城市精细化管理水平不断提高。建设社区公园21个、特色景观街5条，完成困难企业社区环境综合整治18个，新增公园绿地174公顷，城市绿化覆盖率达38.5%。继续实施畅通工程，在公安部、建设部组织的全国“畅行程度”指标评分中居第5位，荣获全国畅通工程模范管理城市称号。

　　四是着力统筹城乡发展。继续推进农村公路通达、电网改造、安全饮水、环境整治“四大工程”建设，新建、改造农村公路1800公里；解决45万人饮水安全问题；完成骨干山塘清淤1万口，增加库容800万立方米；完成农村电网改造投资3.5亿元；完成500头以上规模养殖场污染治理440家；完成农村无害化户厕改造4.2万座。实现粮食生产“七连增”、养殖业产值134.3亿元、农产品加工销售收入560亿元，新增农民专业合作组织630家。完成集体林权制度主体改革，启动县级生态公益林补偿制度，森林覆盖率53.4%。推进四大片11个试验点和以镇带村试点工作，为城乡一体化发展发挥良好示范作用。

　　五是着力加快两型社会建设。先导区建设全面推进，滨江新城、梅溪湖等四大片区开发力度加大，西湖文化园、洋湖湿地公园等重大项目开工建设。加大综合配套改革力度，加强土地规划、储备和耕地保护，有效治理宁乡县大成桥沉陷区地质灾害。积极推进“能评”工作，依法关闭污染企业135家，超额完成省政府下达的SO2和COD减排任务。单位地区生产总值综合能耗、单位规模工业增加值能耗继续下降。环保三年行动计划基本完成，垃圾分选工程一期竣工投产，城市生活垃圾无害化处理率100%；垃圾填埋场渗液深度处理工程投入使用，减少了对周边环境和居民的影响。

　　六是着力解决群众切身利益问题。坚持财力向民生倾斜，投入61.8亿元，全面完成省、市实事工程。大力推进创业富民，新增工商企业约1.5万家，新增城镇就业12.3万人，城镇登记失业率为2.9%。继续推进经济适用房、廉租房建设，新建公租房1800多套。建设居家养老服务中心（站）350个，改造农村危房2000户、国有林场危旧房170户。加强物价监测和菜篮子工程建设，对困难群体发放临时价格补贴。食品安全城市建设取得良好成效。完善健全信访责任体系、联网受理群众信访、联调解决群众诉求、联合办结信访积案的“一健三联”工作机制，妥善解决群众信访问题。针对国企改制后到龄不能退休、部分困难企业职工无医保个人账户、1995年前老工伤等历史遗留问题，安排专项资金进行解决，惠及3万多个家庭。深入推进平安长沙建设，创新社会管理方式，开展“五清查一打击”专项行动。加强应急管理、生产安全、消防安全等工作，较大以上安全事故明显下降。

　　七是着力促进社会事业发展。职教基地建成投入使用，中小学校舍安全工程进展顺利，城乡免费义务教育全面实施。广播电视“村村通”工程全面完成，绿色上网场所基本实现社区全覆盖。文化产业快速发展，实现增加值450亿元，增长20%。群众体育与竞技体育协调发展，4人次获第16届亚运会冠军，荣获省第十一届运动会金牌数、总分第一。加快医药卫生体制改革，率先全国实现城乡居民医疗保险制度并轨，试点实施医疗卫生机构基本药物零差率销售。继续开展引进储备万名优秀青年人才和国际高端人才行动。人口自然增长率为4.4‰，被评为全国人口和计划生育综合改革示范市。统计基础工作进一步加强，人口普查顺利推进。人民防空、国防动员、双拥共建和民兵预备役建设取得新进步。工会、共青团、妇联、科协、文联、残联、侨联、社科联、贸促会、红十字会、关工委、老龄委等为长沙发展做出新贡献。

　　八是着力加强政府自身建设。基本完成新一轮市本级政府机构改革，政府职能进一步转变。坚持依法行政，率先全国开展地方立法听证，加强规范性文件审查，创建政府法制建设白皮书制度。继续开展“两帮两促”活动，解决各类困难问题1215个。推进政府信息公开，完善办事公开制度，优化行政审批流程，取消或暂停实施年检事项53项，取消非行政许可200余项，市级行政许可事项全部实现在线办理。实施经营性国有资产全面监管，确保国有资产保值升值。规范公务接待，从严控制公费出国出境。自觉接受人大和政协监督，办理人大代表建议、批评、意见337件、政协提案308件，满意率分别达99.7%、99%。民族宗教、对台、档案、机关事务、市志、气象、地震、公积金管理、移民等工作取得新成绩。

　　各位代表，2010年是“十一五”的最后一年，经过全市人民五年拼搏，我们圆满完成了“十一五”确定的各项目标任务。

　　五年来，综合实力大幅提升。地区生产总值达到2005年的2.4倍，年均增长15.4%，人均地区生产总值超过1万美元；地方财政收入达到2005年的3.6倍，年均增长29.4%；全社会固定资产投资、社会消费品零售总额年均分别增长28.9%、19.3%，高于预期目标14和5.3个百分点；五年实际直接利用外资87.8亿美元，是“十五”的3.5倍；经济总量在全国省会城市排名第7位，所辖四县（市）在全国县域经济基本竞争力百强中排名大幅前移。

　　五年来，城乡面貌显著变化。围绕打造宜居城市，完成基础设施建设投资1800多亿元，建成区面积由167.7平方公里扩展到256平方公里。建成湘江风光带、橘子洲风景区等一批精品工程，实施地铁、过江通道等一批重大项目，进行大规模棚户区改造。开展文明城市创建，加强城市精细化管理，城市形象明显提升。开展“百村示范、千村整治”和农村“四大工程”建设，农村生产生活条件明显改善，新农村建设和城乡一体化示范工程取得明显成效。

　　五年来，民生社会事业全面发展。努力促进“学有所教、劳有所得、病有所医、老有所养、住有所居”。城乡免费义务教育全面实施；深入开展创业富民活动，新增城镇就业岗位52.6万个，城乡居民人均可支配收入年均分别增长12%和17.9%；全面推行城乡居民基本医疗保险、基本养老保险制度，城乡低保家庭实现应保尽保；推进安居工程，8万多户住房困难家庭搬进新居，城乡居民人均居住面积分别增长2.8平方米和11.4平方米。民族团结进步，宗教关系和谐。文化、体育、人口计生、广播电视、新闻出版等各项事业全面协调发展。

　　五年来，两型社会建设卓有成效。先导区建设全面推进，综合配套改革不断深化，生态环境持续改善。单位地区生产总值综合能耗降低20%，全面完成“十一五”节能减排任务；城市空气质量优良率、污水处理率分别比2005年提高25.5、45个百分点。先后荣获联合国人居环境良好范例奖和国家森林城市、国家园林城市等多项殊荣。

　　各位代表！这些成绩的取得，是市委正确领导的结果，是各级人大、政协监督支持的结果，是全市上下团结奋斗的结果。在此，我代表市人民政府向全市各族人民，向各民主党派、工商联、人民团体和各界人士，向驻长部队、武警官兵和政法干警，向所有关心支持长沙发展的朋友们，表示诚挚的感谢和崇高的敬意！

　　在总结成绩的同时，我们也清醒地看到，长沙在快速发展过程中仍然存在一些不容忽视的问题：调整经济结构、转变发展方式的任务仍然艰巨；受输入型物价上涨的影响，我市居民消费价格指数达到102.9，给人民生活特别是低收入家庭带来不利影响；城市规划执行不严、建设比较粗放、管理欠精细的现象依然存在，提升城市品质仍需付出更多努力；安全事故在个别领域仍有发生，社会治安还存在薄弱环节；服务质量和办事效率与群众的期望还有差距，政府管理和政风建设需进一步加强等。对此，我们深感责任重大，必须采取有效措施认真加以解决。

　　二、关于“十二五”规划

　　根据市委《关于制定长沙市国民经济和社会发展第十二个五年规划的建议》，市政府制定了《长沙市国民经济和社会发展第十二个五年规划纲要（草案）》，提请大会审议。

　　总体判断，“十二五”时期，长沙正处于可以大有作为的机遇期、率先实现小康的决战期、转变发展方式的攻坚期和奠定城市格局的关键期。我们既面临良好的机遇，也面临巨大的挑战。

　　一是发展环境总体趋好，但存在很大不确定性。世界经济总体呈现温和复苏态势，我国应对危机的有效措施，将促进经济持续平稳发展。但近期美国实施量化宽松货币政策，欧元区主权债务危机还在发展，引发全球货币流动性泛滥，带动资源性产品价格上涨，给未来经济增长带来了不确定性，不可避免地对我国经济发展、进出口贸易和物价水平带来不利影响，增加了输入型通胀风险，国家的财政、金融、土地等宏观政策也可能随着形势的变化而适时调整。我们必须准确把握形势，科学应对变化。

　　二是经济总量持续增长，但转变发展方式任重道远。“十一五”期间，长沙经济总量大幅增长，综合实力明显增强，但发展方式相对粗放：经济增长过分依赖投资拉动；具有自主创新能力和核心竞争力的大企业相对较少；优势产业不多，部分产业处于价值链低端；新型服务业态和现代农业发展相对滞后。而应对金融危机和改善民生、维护稳定需要我们加快发展。我们面临加快发展与转变方式的双重压力，迫切需要在加快发展中大力转变发展方式。

　　三是城市快速发展，但资源环境要素保障任务艰巨。“十一五”期间，长沙城市面积和人口迅速增加，“十二五”期间这种快速发展的趋势仍将延续。但目前城市发展主要是外延式扩张，土地需求旺盛，部分区县（市）和园区土地资源消耗过快；煤、电、油、气等要素基本依赖输入，足额保障困难。国家对土地、环境保护、节能减排提出了更高更严的要求，破解这些瓶颈，必须按照两型社会建设的要求，注重集约节约发展，减少对资源环境的影响。

　　四是民生不断改善，但与群众期待仍有差距。“十一五”期间，我市民生社会事业全面进步，人民群众普遍享受改革发展成果。但随着社会更加开放、信息日益透明，人民群众对民生改善有更高的期待。目前，加快经济发展、加大基础设施建设的投入仍然很大，有限的财力既要保民生，又要为未来发展增后劲，改善民生的愿望与有限的财力矛盾仍比较突出。

　　五是发展优势明显，但区域竞争更加激烈。长沙毗邻珠三角和长三角，随着武广高铁开通，沪昆高铁、城际铁路建设，长沙将成为全国重要的交通枢纽城市，区位优势更加明显。同时，长沙良好的教育、科技、人才、生态优势，有利于引进战略投资者，承接高端产业转移。但各地城市发展你追我赶，东部沿海城市抢先转型发展，近年被我们赶超的城市与我们差距不大，我们稍有松懈就会被反超；一些位居我们后面的城市发展更快，随时有可能赶超我们，必须增强发展的危机感和紧迫感。

　　六是行政能力普遍提高，但与新形势的需要不相适应。近年来，我们大力推进政府精细化管理，加强公务员队伍建设，各级政府公共管理服务水平大为提高。但我们正步入一个利益多元化的时代，不同的利益主体有不同的利益诉求；社会结构发生很大变化，城乡互动更加紧密，新经济组织蓬勃发展，群众民主法制意识增强，一些深层次矛盾错综复杂，对社会管理提出了巨大挑战，不断考验我们的行政能力。政府工作人员的视野、专业素养、敬业精神、服务水平需要进一步提高。

　　基于以上分析，总体来看，未来五年我们的机遇大于挑战，只要我们发挥优势，扬长避短，就能抢占先机，实现新的跨越。

　　按照市委提高城市国际化水平、加快产业高端化进程、推进城乡一体化发展、创新发展两型化模式、加强城市法治化管理的总体要求，围绕率先基本建成两型城市和实现全面小康的总体目标，坚持以人为本、两型引领、创新驱动、城乡统筹、协调发展原则，到2015年，地区生产总值年均增长13%；财政总收入年均增长15%；全社会固定资产投资年均增长18%；社会消费品零售总额年均增长18%。城乡居民人均可支配收入年均分别增长12%和14%；城镇登记失业率控制在4%以内，社会保险覆盖率达95%以上，价格总水平基本稳定；单位地区生产总值综合能耗降低15%，主要污染物排放总量减少10%；人口自然增长率控制在5.7‰以内。

　　上述目标中，既有定性目标，又有定量指标，既有预期性目标，又有约束性指标，既系统考量了“十二五”经济社会发展面临的宏观环境，又充分考虑了全市人民过上更加美好生活的新期待，体现了战略性、前瞻性、指导性的要求。为此，我们要坚定不移地把长沙建设成为具有国际视野、人民引以自豪的创业之都、宜居城市、幸福家园。

　　----发展方式深刻转变，产业结构更加优化，城乡经济繁荣发达，经济总量和综合实力跻身省会城市前列。创业氛围更加浓厚，创业环境日益优越，创业主体活力迸发，成为具有国际竞争力的创新创业城市。

　　----城市配套功能完善，交通出行方便快捷，景观建筑富有魅力。人与自然和谐共处，山水洲城风貌凸显，人居环境更加舒适，成为全国生态文明示范城市。

　　----城乡居民收入大幅增长，社会保障水平显著提升，基本公共服务均等化发展，人民生活更加殷实。利益协调机制全面构建，社会治安环境安定有序，人际关系更加和谐，成为社会公平正义、发展成果共享的幸福家园。

　　为确保“十二五”各项目标任务的顺利完成，未来五年，我们必须着力抓好五大领域的工作：

　　（一）大力推进经济建设，促进经济平稳健康发展。推动经济结构战略性调整。增强居民消费能力，优化投资结构，大力发展外向型经济，促进消费、投资与出口协调发展。推动自主创新。实施人才强市战略和知识产权战略，加强产业技术创新，推进科技成果转化，把长沙建设成为全国重要的科技创新中心和科技成果转化中心，建设国家创新型城市。推动产业高端化发展。培育战略性新兴产业，改造提升传统产业，大力发展现代服务业和现代农业，促进三次产业结构由“二三一”向“三二一”转变。

　　（二）大力推进城市建设，加快城市国际化进程。完善城市功能。建设以综合交通为主导，以现代化市政基础设施为支撑的城市国际化基础平台；加快“数字城市”建设，促进“三网融合”发展。提升城市品质。加大片区开发、棚户区改造和“城中村”改造力度，加强城市综合体、精品小区、两型小区和智能小区建设，精心雕琢城市细节，努力建设现代宜居城市。深化对外开放。加强全方位、多层次对外交流合作，提升引资引智水平；实施“走出去”战略，推动外向型经济发展，提高城市国际化水平。

　　（三）大力推进两型社会建设，创建生态文明城市。加强资源节约和生态环境保护。深入开展节能工作，大力开发利用新能源和可再生能源，建立健全资源节约标准体系、技术体系和管理体系。加强城乡生态环境保护和建设，全面形成城市、森林、农田、湿地、流域等五大生态体系。深化综合配套改革。在资源环境管理制度、城乡统筹体制机制、土地集约节约利用、政府管理创新转型等方面率先突破，探索形成两型社会建设体系。加快先导区建设。创新区域发展方式、城乡建设模式和行政管理体系，实现起步区发挥效应、核心区基本建成、规划区形成功能，率先建成具有示范作用的两型新城。

　　（四）大力推进新农村建设，实现城乡统筹发展。加快城市基础设施向农村延伸。加大以水利为重点的农村基础设施建设投入，强化城乡基础设施衔接配套，逐步实现供水、污水处理、公交、燃气、信息等基础设施乡镇集镇全覆盖。加快城乡产业融合。统筹城乡产业发展规划，调整城乡产业布局，加快工业与现代农业、现代农业与服务业的融合，发展壮大县域经济。加快新型城镇化进程。优化城镇结构，形成主城区、都市圈、卫星城镇层次分明、组合有序的城镇体系，推进农村社区化、农民市民化。

　　（五）大力推进社会建设，促进基本公共服务均等化。健全社会保障体系。深入推进创业富民，努力扩大就业，提高居民收入水平。加快社会保险和社会救助体系建设，发展社会福利和慈善事业，加强保障性住房建设，构建城乡一体的社会保障体系。推动社会事业全面发展。优化教育资源配置，推进教育强市战略。积极推进医药卫生体制改革，大力开展全民健身，推进食品安全城市建设。加强历史文化和非物质文化的保护传承，推进现代文化创新发展，建设国际文化名城。提高社会管理服务水平。推进社会管理创新，加强社会治安综合治理，加快社会法治化进程，健全公共安全体系，积极培育社会工作人才，增强社会管理能力。

　　在工作实践中，需要更加注重解决以下几个事关长沙科学发展的关键性问题：

　　（一）更加注重科技创新。加快转变发展方式当前最紧迫的是推动科技创新。在资源环境约束加剧、产能总体过剩的情况下，经济发展必须依靠科技进步，抢占制高点，实现人无我有、人有我优，在激烈的竞争中立于不败之地。必须大力培养和引进人才，加大科技创新和成果转化力度，培育发展战略性新兴产业，改造提升传统产业，促进发展方式转变。

　　（二）更加注重区域消费平台建设。中心城市具有大力发展服务业的优势和空间。坚持差异化发展战略，以区域消费平台建设推动消费转型升级，着力建设“一站式”大型商业综合体和大型商务中心；整合医疗、教育资源，打造区域医疗、教育中心。加快发展金融、保险、旅游等现代服务业，引进国际品牌，吸引高端消费，增强长沙消费的辐射力与影响力。

　　（三）更加注重城市集约发展。城市品质和建设时序至关重要。坚持以国际视野、国际标准规划建设城市，打造精品工程，提高城市品质。按照“沿江建设、跨江发展”战略，有序推进“一江两岸”和高铁、地铁、城际轨道沿线，省府新区、黎托新区、南湖片区、金霞片区、空港新区及先导区滨江新城、梅溪湖、洋湖垸、滨水新区等片区开发建设，集约节约用地，完善基础设施配套，形成集聚效应，打造长沙经济新的增长极。

　　（四）更加注重综合交通建设。通畅便捷的城市交通是宜居城市的重要标志。目前，长沙机动车以每年20万辆以上的速度递增，未来五年的城市交通面临更大挑战。必须以综合交通的理念破解城市交通问题。坚持公交优先发展，引导机动车有序增长，鼓励绿色出行。加快地铁、过江通道及主要干道等交通设施建设，优化路网，打通瓶颈，完善配套，发展智能交通，构建“全面覆盖、衔接顺畅、运行高效”的城乡交通运输体系。

　　（五）更加注重改善民生。改善民生是政府一切工作的出发点和落脚点。必须把群众呼声作为决策基础，把群众需要作为工作导向，倾注更多精力，集中更多财力，最大程度满足不同层次群众的合理需求。着力解决就业、就学、就医、社保等群众现实利益问题，特别是重点解决城市中低收入家庭住房困难，从根本上提高群众的满意度，实现发展指标与幸福指数同步提升。

　　（六）更加注重政府自身建设。完成繁重的发展任务，应对多变的复杂局面，需要不断加强政府建设，提高行政能力。始终坚持依法行政，突出规范行政执法行为，建设一支高素质的公务员队伍。始终坚持民主决策、科学决策，减少决策失误。大力推行精细化管理，坚持勤俭办一切事业，厉行节约。加强与公众沟通，完善政府常务会议市民代表旁听等制度，搭建政府和公众互动交流平台，融洽政府与群众关系，树立政府良好形象。

　　三、2011年主要任务

　　2011年是继往开来、实现新跨越的关键一年，也是既复杂又困难的一年。今年经济社会发展主要预期目标是：地区生产总值增长13%；财政总收入增长15%；全社会固定资产投资增长20%；社会消费品零售总额增长18%；单位地区生产总值综合能耗下降3%，主要污染物排放总量减少2%；城乡居民人均可支配收入分别增长12%和14%；城镇登记失业率在4%以内；人口自然增长率在5.7‰以内；居民消费价格指数控制在104左右。

　　为此，我们要全面贯彻科学发展观，认真落实党的十七届五中全会和中央、省委、市委经济工作会议精神，突出抓好以下几个方面的工作，努力实现“十二五”良好开局。

　　（一）加快重大项目建设。突出重大项目带动。重大项目建设是稳增长、增后劲的战略举措。着力推进沪昆高铁、石长铁路复线、城际铁路、长浏高速、长韶娄高速等中央、省在长建设项目。加快轨道交通、湘江综合枢纽、大河西综合交通枢纽等重大工程建设。抓好中联重科、三一重工扩产和广汽菲亚特、比亚迪等重大产业项目建设。力争全年实施重点项目463个，完成投资1848亿元。加强项目要素供给。密切与国家、省相关部门的汇报衔接，争取更多项目进国家和省项目库。进一步推动银企合作，保持信贷规模稳定增长。推进先导控股、城投集团、轨道集团融资功能改造，鼓励企业上市融资，着力激活民间投资。优化土地供应调控，确保重点建设项目用地需求。加强煤、电、油、运等生产要素保障。强化项目管理服务。完善重点项目遴选机制，加强项目前期工作。牢固树立全寿命周期成本最小理念，以建设质量提升投资效益。严格项目建设全程监管，严肃查处违法违规问题。继续开展“两帮两促”活动，提升为项目、为企业服务水平。

　　（二）加快新型工业化步伐。提升自主创新能力。推进区域创新体系建设，加强知识产权保护。实施一批重大科技专项，加强国际先进适用技术、共性技术的引进和创新，提升企业关键技术研发、核心部件制造和品牌培育能力。继续实施引进国际高端人才和储备万名优秀青年人才计划，完善人才引进后续管理。推进产业集群发展。完善战略性新兴产业规划，培育发展新材料、生物医药、电子信息、文化创意等战略性新兴产业。做大做强装备制造、汽车及零部件等优势支柱产业，改造提升食品烟草、烟花鞭炮等传统特色产业。建立中小企业融资服务中心，促进中小企业发展。支持一批建筑企业做大做强。鼓励扶持有条件的企业对外投资发展，参与国际分工合作。加快园区提质改造。优化园区功能定位和产业布局，进一步理顺园区管理体制。发展工业地产，加快园区标准化厂房建设。推进高新区创新型科技园区建设，支持经开区扩规发展，建设国家级新型工业化产业示范基地。

　　（三）加快现代服务业提质。推进消费转型升级。着力建设高端消费平台，推进万达广场、保利南湖广场、东牌楼等商业综合体建设。支持一批“中华老字号”企业做大做强。大力发展会展经济，培育娱乐、购物、旅游等消费品牌，加快铜官窑遗址公园、灰汤温泉、岳麓山景区、大围山景区等精品旅游项目建设，实现旅游总收入550亿元以上。推进“万村千乡”市场工程建设，继续实施农贸市场提质改造。促进现代服务业融合发展。重点发展现代物流、电子商务、服务外包、物联网等新型服务业，推动服务业与制造业、信息化与工业化深度融合。大力发展金融业，推进区域性金融中心建设，支持长沙银行打造成为区域性银行。加快现代服务业集聚区发展，推进芙蓉中央商务区建设。扩大开放合作。重点引进世界500强、跨国公司、中央国企、金融机构等大型企业集团，实现直接利用外资增长15%。着力培育出口龙头企业，积极申报国家级工业装备出口创新基地和花炮出口创新基地，支持金霞保税物流中心创建综合保税区。

　　（四）加快现代宜居城市建设。坚持科学规划引领。完成城市总体规划修改，优化功能布局。强化规划执行刚性，严格规范用地性质和容积率调整。深入推进规划进社区工作，抓好规划展示馆布展。完善城市基础设施。推进“一江两岸”建设，加快湘府路大桥、福元路大桥、南湖路隧道等过江通道建设，实现潇湘大道、湘江大道城区段和营盘路隧道建成通车。突出省府新区、浏阳河风光带、新河三角洲、黎托新城等片区开发建设。继续实施交通疏导工程，畅通交通微循环，规范城区停车。优先发展公共交通，加快公交车辆更新，加强城市快速公交网络和枢纽站点建设。推进国际文化名城建设。加快公共文化服务体系建设，加大历史文化名城和非物质文化遗产保护力度，加强文化市场管理，支持文化主导产业发展，深化文化体制改革。加快国际化城市公共标识建设，启动城区路名路牌更新改造。基本建成“两馆一厅”。争创全国文明城市。大力推进人民满意城市建设，积极开展市民巡防、文明劝导等文明创建活动。规范窗口行业服务行为，提升市民文明素质。开展市容环境、违章建筑、交通秩序等专项整治，提高城市数字化管理水平。推进“城中村”改造试点，完成30个社区公园和5条景观街建设。对开放式社区全面推行物业服务，打造一批精品示范社区。加强城市林业生态圈建设，城市空气质量优良率保持在92%以上。力争成为全国文明城市。

　　（五）加快城乡一体化进程。强化城乡基础设施对接。抓好金洲大道西延线、开元东路东延线等干线公路改造。继续推进农村“四大工程”建设，新增硬化通村公路1500公里，加强农村公路养护；新解决30万农村人口饮水不安全问题；新建沼气池8万立方米；新建和改造一批小型水利工程。关心、支持边远乡镇发展。积极发展现代农业。科学规划优势农业区域布局，支持特色农业发展，大力发展农产品加工业。实行动物免费强制防疫，建立农产品质量可溯制度和市场准入制度，抓好农业标准化、设施化基地建设，重点发展蔬菜产业种苗基地、示范园。提高农业保险水平，增强农业抵御风险能力。促进农民持续增收。落实强农惠农政策，促进农业增产增效，增加农民生产收益。发展农村职业教育，开展农民技术培训，继续挖掘务工增收潜力。扶持农民工返乡创业，提高农民就业创业能力和致富带动能力。

　　（六）加快大河西先导区发展。着力提升承载功能。加快骨干道路建设，实现坪塘大道等8条道路建成通车。加大配套设施建设力度，启动雷锋配水厂主体工程、二水厂扩容工程建设，完成龙王港、洋湖等输变电工程和燃气次高压管线工程。加快生态修复和环境建设，提升环境承载功能。突出重点片区发展。推进梅溪湖、滨江新城、洋湖垸等片区建设，加快滨江新城商业综合体、梅溪湖国际文化中心、洋湖垸城市中心广场等重大项目建设，建成梅溪湖湖泊工程、“两山一湖”二期整治工程等重点项目，启动麓谷新区、坪塘片区、高星片区、滨水新区建设。加快发展两型产业。淘汰落后产能，推进污染企业和核心区内矿山退出，扶持重大产业项目发展，着力打造高端产业集聚区。支持国家超级计算长沙中心、中电软件园等建设，启动大河西人才创业示范区建设，搭建高层次人才创业平台，加快构建长沙发展新引擎。

　　（七）加强综合配套改革。促进资源集约节约利用。制定落实园区项目用地规模和投入产出控制指标，节约集约用地。健全节能管理体系，加强重点领域和全民节能，加强固定资产项目节能评估和审查，推进节水型试点城市和可再生能源建筑应用示范城市建设。加大环境保护力度。实施新一轮“环保三年行动计划”，持续开展大气污染防治和湘江流域长沙段水环境综合整治，加强污泥和餐厨垃圾处理。推进农村环境综合整治，加快乡镇污水处理厂、垃圾收集处置工程建设，继续开展畜禽养殖污染治理，加大株树桥水库和城乡饮用水源地保护力度。突出重点领域改革创新。推进人口户籍、农村土地、农村金融等重点改革，继续开展排污权有偿使用和交易。深化集体林权制度配套改革，推进林权抵押贷款和森林保险试点。建立补充耕地储备库和城乡建设用地增减挂钩库，构建农村土地利用指标交易机制。

　　（八）加强民生保障。争创全国首批创业型城市。深入开展创业富民工程，完善城乡统筹的创业、就业扶持体系。提高职业技能培训水平，建立健全工资增长、集体协商和支付保障等机制，新增城镇就业10万人以上，保持城镇零就业家庭动态清零。提高社会保障水平。扩大社会保险覆盖面，逐步将各类人员纳入参保范围。进一步完善城乡居民基本养老保险和医疗保险制度，实现农村“五保”对象住院医疗费用全额减免。规划建设区县（市）社会福利中心，实施新一轮农村敬老院改扩建。完善社会救助体系，发展慈善公益事业，做好红十字会和移民工作。积极应对价格上涨。物价问题直接关系群众生活。要尽最大的努力减少市域性上涨因素，减少对低收入家庭的影响。实施新一轮菜篮子工程，加强价格监管，着力稳定重要农副食品价格。完善补贴制度，健全最低生活保障、失业保险标准与物价联动机制，妥善安排好低收入群众生活。实施安居工程。继续推行经济适用住房货币补贴，加快发展公共租赁住房。推进黄兴北路、火车北站二期等城市棚户区和5712厂、重型机器厂、九尾冲等国有工矿棚户区改造。加快公共租赁住房建设，新建（筹集）廉租房7000套。继续实施农村危房和国有林场危旧房改造。

　　（九）加强社会建设。优先发展教育事业。理顺教育管理体制，优化教育资源布局，打造一批教育品牌。完成中小学校舍安全工程，启动义务教育学校标准化建设，着力缓解“择校”矛盾。规范发展学前教育，推进示范性职业院校建设。加快健康城市建设。加强食品药品安全监管，深入推进食品安全城市建设，实施放心猪肉、放心蔬菜、放心作坊、放心街市示范工程。优化卫生、体育设施布局，逐步整合卫生和计生妇幼保健资源、社会体育和学校体育资源。深入推进医药卫生体制改革，加大重大传染疾病预防控制，加强基层医疗服务队伍建设。广泛开展全民健身运动，筹备参加第七届全国城市运动会。加快市老干部活动中心建设和市青少年宫改建。推进社会管理创新。全面推行“一健三联”信访机制，加强人民调解工作，妥善处理群众信访突出问题。关心社区基层干部，推进社会工作者队伍建设。完善应急管理体系，创建全国综合减灾示范社区。加强网络信息和社会心理服务工作，提升流动人口服务管理水平。注重治安防控体系建设，构建预防、打击犯罪新机制，及时解决突出的治安问题。建设和谐长沙。强化计划生育基层基础和综合治理工作，统筹解决人口问题。继续抓好第六次人口普查。落实安全生产责任制，加强消防安全，强化人口密集区和重大危险源安全管理。依法管理宗教事务，促进民族团结进步。切实保护妇女、儿童、老年人、残疾人的合法权益。加强国防动员、教育和后备力量建设，争创全国双拥模范城五连冠。

　　（十）加强和改进政府管理。推进依法行政。完善重大决策专家咨询论证和合法性审查，推行立法后评估制度，严格规范性文件制定审核，加强行政复议应诉工作。推进政府精细化管理，实行公共财政、政府投资、资源资产全程监管。规范政府合同管理。打造阳光政府。贯彻落实《政府信息公开条例》，不断增强政府工作透明度；继续优化行政审批流程，提高行政效能。自觉接受人大法律监督、工作监督和政协的民主监督，认真执行人大常委会的各项决定、决议，及时办理人大代表议案、建议和政协提案。加强与民主党派、工商联、人民团体和社会各界人士的联系。加强监察、审计监督，主动接受社会和新闻舆论监督，让人民更有效地监督政府。加强政风建设。大兴求真务实之风，坚持从群众需要出发、从发展需要出发想问题、办事情，全面完成省、市实事工程。严肃政令，强化政务督查，提高执行力。加强基础管理，完善公共信息和技术服务平台建设。强化绩效考核，严格行政问责和行政过错责任追究。建立健全教育、制度、监督并重的惩治和预防腐败体系，严厉查处腐败案件。注重提升公务员专业知识和专业素养，加强公务员队伍的教育、管理和监督，努力建设一支人民满意的公务员队伍。

　　各位代表！站在新的历史起点上，我们已经踏上率先基本建成两型城市和实现全面小康的新征程，肩负着科学发展、和谐发展、率先发展的光荣使命。让我们在市委的坚强领导下，紧紧团结和依靠全市人民，把握机遇，开拓创新，携手共进，共同开创“十二五”发展良好开局，以优异成绩向建党90周年献礼！
